# The Use of the Masquelet Technique in the Treatment of Pathological Distal Third Femoral Fracture Secondary to Chronic Osteomyelitis

**DOI:** 10.3390/life15040537

**Published:** 2025-03-25

**Authors:** Razvan Cosmin Tudor, Daniel Vasile Timofte, Norin Forna, Oana Viola Badulescu, Liliana Savin, Tudor Pinteala, Dan Mihailescu, Tudor Ciobotariu, Alin Ciobica, Mihnea Theodor Sirbu, Paul Dan Sirbu, Serban Dragosloveanu, Bogdan Sorin Capitanu, Romica Cergan, Cristian Scheau

**Affiliations:** 1 Department of Orthopedics and Traumatology, Surgical Science (II), Faculty of Medicine, “Grigore T. Popa” University of Medicine and Pharmacy, 700115 Iasi, Romania; 2 “Dr. Iacob Czihac” Military Emergency Clinical Hospital, 700483 Iasi, Romania; 3 Department of Surgery, “Grigore T. Popa” University of Medicine and Pharmacy, 700115 Iasi, Romania; 4 “Sfantul Spiridon” Emergency County Clinical Hospital, 700111 Iasi, Romania; 5 Clinical Hospital of Medical Rehabilitation, 700661 Iasi, Romania; 6 Department of Pathophysiology, Morpho-Functional Sciences (II), Faculty of Medicine, “Grigore T. Popa” University of Medicine and Pharmacy, 700115 Iasi, Romania; 7 Faculty of Medicine, “Grigore T. Popa” University of Medicine and Pharmacy, 700115 Iasi, Romania; 8 “Ioan Haulica” Institute, Apollonia University, 700511 Iasi, Romania; 9 Department of Biology, Faculty of Biology, Alexandru Ioan Cuza University of Iasi, 700506 Iasi, Romania; 10 Center of Biomedical Research, Romanian Academy, 700506 Iasi, Romania; 11 Academy of Romanian Scientists, 050094 Bucuresti, Romania; 12 Department of Orthopaedics and Traumatology, The “Carol Davila” University of Medicine and Pharmacy, 050474 Bucharest, Romania; 13 Department of Orthopaedics, “Foisor” Clinical Hospital of Orthopaedics, Traumatology and Osteoarticular TB, 021382 Bucharest, Romania; 14 Department of Anatomy, The “Carol Davila” University of Medicine and Pharmacy, 050474 Bucharest, Romania; 15 Department of Radiology and Medical Imaging, “Foisor” Clinical Hospital of Orthopaedics, Traumatology and Osteoarticular TB, 021382 Bucharest, Romania; 16 Department of Physiology, The “Carol Davila” University of Medicine and Pharmacy, 050474 Bucharest, Romania

**Keywords:** segmental defects, Masquelet technique, osteomyelitis, pathological fracture, treatment, imaging

## Abstract

Introduction: Chronic osteomyelitis is a persistent infection of the bone that poses significant challenges, particularly when associated with pathological fractures and extensive bone defects. This case report highlights the application of Masquelet’s induced membrane technique (MIMT) in managing a complex distal femur defect in a 50-year-old male with a long-standing history of chronic osteomyelitis. The patient presented with a non-union fracture, severe osseous destruction, and infection, requiring a multidisciplinary approach. Case report: The first stage involved radical debridement of necrotic tissue and stabilization with a titanium plate and an antibiotic-impregnated polymethylmethacrylate spacer to induce a bioactive membrane. The second stage, performed 30 days later, after infection resolution, entailed removing the spacer, harvesting an autologous iliac crest bone graft, and filling the defect within the preserved membrane. Postoperative care included a tailored antibiotic regimen and gradual weight-bearing, leading to favorable clinical and radiological outcomes. Conclusions: This case demonstrates the utility of MIMT in reconstructing extensive bone defects while addressing infection. The technique provides a reliable and effective alternative to amputation, offering high success rates and functional restoration in complex cases.

## 1. Introduction

Chronic osteomyelitis is a long-lasting or recurrent infection of the bone and surrounding tissues, usually caused by bacteria, which can develop after trauma or surgery, through hematogenous spread, or as a result of chronic wounds. This condition is associated with various complications, including sepsis, chronic disability, pathological fractures, and, in severe cases, amputation [1,2,3,4].

Although it is a rare complication, managing bone fractures in the context of chronic osteomyelitis presents significant challenges due to the interplay of persistent infection, bone fragility, and impaired healing. These issues can lead to segmental bone defects, requiring an aggressive and multidisciplinary treatment approach to achieve optimal outcomes [5,6,7,8,9].

Segmental bone defects present complex challenges and are associated with significant long-term morbidity. Historically, managing extensive segmental bone defects was highly challenging, making amputation the treatment of choice. However, depending on the size of the defect, a variety of methods can now be employed, including autologous bone grafting, allograft bone transplantation, vascularized fibular graft transfer, distraction osteogenesis (the Ilizarov technique), acute shortening, 3D printed scaffold combined with growth factors, and the induced membrane technique (the Masquelet technique) [10,11,12,13].

Traditional bone grafting techniques often face limitations, such as uncontrolled graft resorption, even when the recipient site is adequately vascularized [14]. On the other hand, the Ilizarov technique, despite its effectiveness, has notable disadvantages. These include a prolonged reconstructive period, which is dependent on the length of the defect being treated, a high complication rate associated with extended use of external fixation, and the significant cost of the ring or spatial external fixation frame [11,15,16,17].

The Masquelet technique is an alternative method for managing segmental bone defects, first described by Masquelet and Begue. It is particularly effective for defects larger than 5 cm (ranging from 5 to 24 cm) where bone autografts alone are insufficient. This procedure involves two operative stages [18]. In the first stage, radical debridement is performed, and a polymethylmethacrylate (PMMA) spacer is temporarily placed to induce the formation of a bioactive membrane at the defect site. The bone defect is then stabilized using either external or internal fixation. In the second stage, typically from 6 to 8 weeks later, once soft tissue healing has occurred, the spacer is removed while preserving the bioactive membrane. The cavity is filled with morselized cancellous bone autograft from the iliac crest. For larger defects requiring more material, bone substitutes may be combined with cancellous bone [11,19].

The purpose of this case report is to share our experience using the Masquelet technique to manage a pathological in chronic osteomyelitis. This approach was chosen for its ability to induce a bioactive membrane, support vascularization, and prevent graft resorption. The case highlights key challenges such as infection control and bone stability, providing insights into the indications, benefits, and limitations of this technique in complex limb reconstruction.

## 2. Case Report

The case involves a 50-year-old Caucasian male who presented to our clinic with a non-union of the left femur, resulting from a pathological fracture in an osteomyelitic bone. The fracture was treated non-surgically, but healing was not achieved. The patient has a history of chronic osteomyelitis, which began at the age of 11 following an injury to the area. Unfortunately, the patient was unable to provide further details regarding this incident.

**Figure 1:**
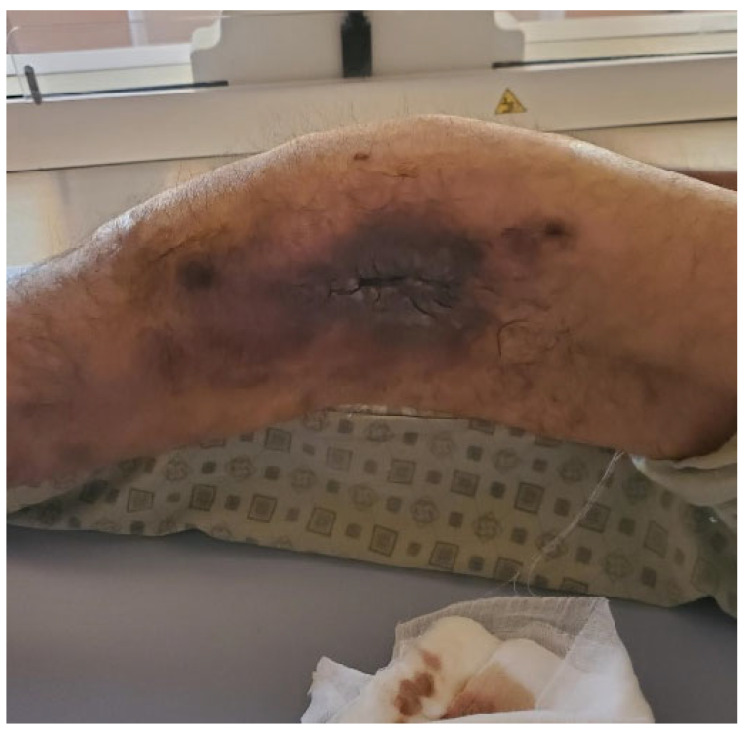
Clinical aspect of the left thigh.

At the time of admission, the physical examination revealed a marked deformity of the thigh, with a necrotic area on the outer side of the left thigh. There is a skin defect approximately 4–5 cm in size, with irregular edges and atonic tissue, along with the secretion of a serosanguinous fluid (Figure 1).

The blood test showed signs of an inflammatory syndrome, with a white blood cell count (WBC) of 12.27 × 10^3^/µL (Normal range: 4.0–10.0 × 10^3^/µL), C-reactive protein (CRP) of 33 mg/L (normal range: 0–5.0 mg/L), fibrinogen level of 6.87 g/L (normal range: 2.0–4.0 g/L), erythrocyte sedimentation rate (ESR) of 130 mm/h (normal range: 0–15 mm/h), platelet count (PLT) of *909 ×* 10^3^/µL (normal range: 163–337 × 10^3^/µL), and hypochromic anemia, with a hemoglobin (Hb) level of 7.8 g/dL (normal range: 13.5–18.0 g/dL) and a mean corpuscular hemoglobin concentration (MCHC) of 31.3 g/dL (normal range: 32.3–36.5 g/dL).

Bacterial testing conducted on the serosanguinous fluid secretion revealed bacterial growth of *Staphylococcus aureus* (MSSA) and *Enterobacter* spp. The specimens were sensitive to all antibiotics tested, with the exception of ampicillin, to which *Enterobacter* spp. showed resistance.

**Figure 2:**
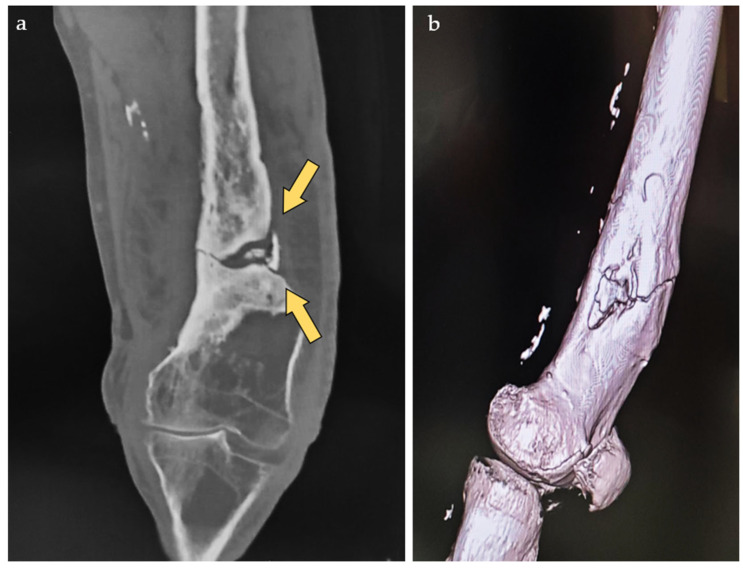
Preoperative CT scan with reformatting in the coronal plane (**a**) and virtual rendering technique (**b**) depicting the non-united fracture (yellow arrows).

Radiographs and computed tomography (CT) scans were performed, revealing a non-united fracture and a sequestrated portion of the femur approximately 20 cm in length (Figure 2).

Despite these challenges, a multidisciplinary team was convened, including specialists from anesthesiology, infectious disease, cardiology, and orthopedics, to develop a rigorous and careful strategy. After reaching an agreement, a plan was devised, and the decision was made to proceed with surgery for the patient. The selected approach was to perform the Masquelet technique and attempt to preserve the patient’s limb, given his youth and demanding functional needs.

**Figure 3:**
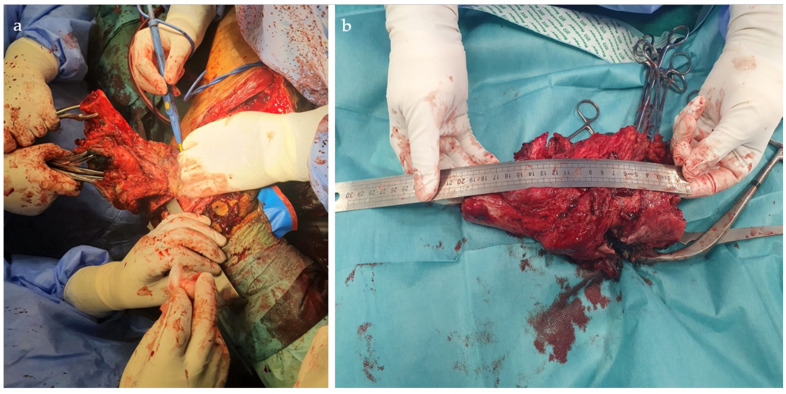
Radical debridement with the removal of all infected or non-viable bone (**a**); Resection Specimen (**b**).

The first surgical stage of the Masquelet technique consists of four main steps: debridement and specimen excision, stabilization, spacer placement, and soft tissue coverage. During the initial step, an approach was made to the external facet of the left thigh, with the skin defect centered around the incision. Following superficial dissection and careful hemostasis, it was noted that the soft tissues adjacent to the affected bone were intensely red with blackish areas, indicating extensive necrosis. The bone displayed an irregular surface with osteolytic signs and patchy sclerosis, indicative of chronic osteomyelitis with advanced osseous destruction. A rigorous debridement of all infected or non-viable bone, as well as the interposed fibrous tissue, was performed, with the excision margins extended until healthy bone was reached. As a result, a 20 cm resected specimen was obtained (Figure 3). Intraoperative samples were collected and sent to the microbiology department, but the results returned negative.

**Figure 4:**
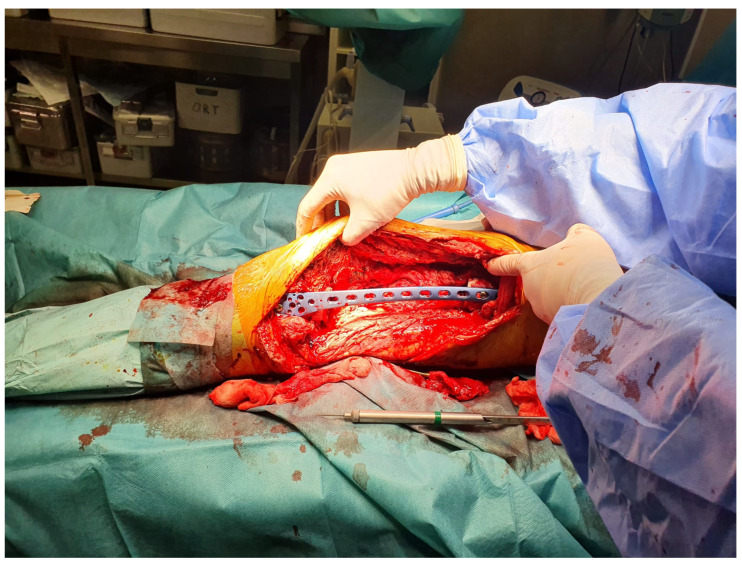
External fixation using a titanium plate.

After debridement and excision, the second step involves stabilizing the limb to maintain length, alignment, and positioning prior to the insertion of the cement spacer. The choice of osteosynthesis method depends on the location of the defect. While an external fixator is typically used for stable fixation in most cases, a titanium plate was chosen in this instance due to the specific characteristics of the case (Figure 4).

**Figure 5:**
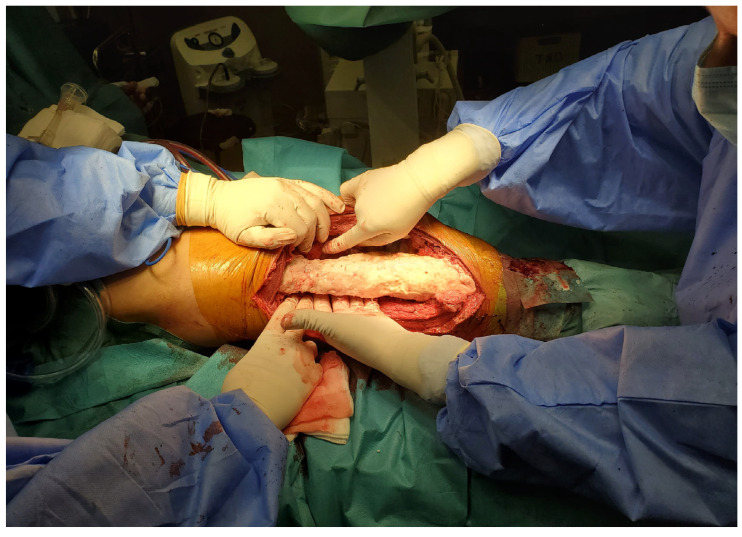
Placing the antibiotic-impregnated cement spacer.

The third step, following stabilization, involves placing the PMMA antibiotic-loaded spacer. The PMMA spacer used in this case was prepared with GENTAFIX^®^ 1 cement, (Teknimed, Montredon L’Union, France). This spacer helps prevent soft tissue collapse into the bone defect and preserves the space needed for bone reconstruction. The cement spacer should fill the intramedullary canal and extend to the edges of the surrounding viable bone. The plate was also covered with cement to prevent biofilm formation and ensure complete stability (Figure 5).

Although the original Masquelet technique does not routinely recommend antibiotic-loaded spacers, their use is supported in cases of chronic osteomyelitis to reduce the risk of persistent infection. Despite negative intraoperative cultures, preoperative microbiological tests confirmed the presence of MSSA and *Enterobacter* spp., justifying the addition of the PMMA antibiotic-loaded spacer. Literature suggests that antibiotic-loaded PMMA spacers help prevent biofilm formation and reduce bacterial load, particularly in cases with a history of infection [20,21]. Given the high risk of recurrence in this patient, the decision to incorporate antimicrobials was intended to improve infection control and overall treatment outcomes.

The absence of signs of local infection at 4–6 weeks suggests that the membrane has formed and the site is ready for grafting.

The fourth and final step is soft tissue coverage and wound healing. The least technically demanding strategy is selected to effectively cover soft tissue defects, which may include advanced wound care treatments or flap coverage procedures, ensuring adequate coverage of the skin defect.

**Figure 6:**
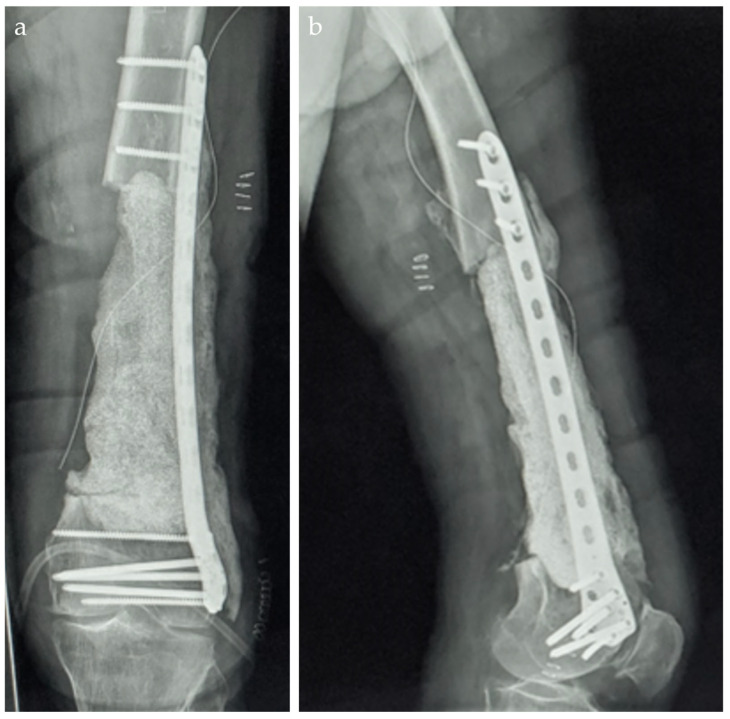
Postoperative Rx aspect. (**a**). antero-posterior view. (**b**). left lateral view.

In the postoperative period, the patient was provided with a series of recommendations and treatment instructions. The weight-bearing load on the operated lower limb is determined by factors such as stability, defect size, location, and the type of implant used. Antibiotic therapy was prescribed for eight weeks: Vancomycin 4 g/day and Gentamicin 240 mg/day for the first 3 days, followed by Sumetrolim 4 tablets/day and Ciprinol 8 g every 12 h. This approach allows adequate time for several critical processes to occur, including the revascularization of the viable marginal tissue surrounding the bone defect, the formation of the self-induced periosteal membrane, and the treatment of any residual infection with systemic and/or local antibiotics. The radiographic aspect is depicted in Figure 6.

**Figure 7:**
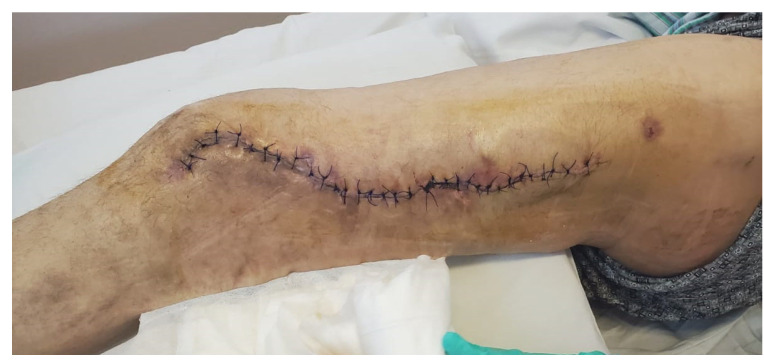
Clinical aspect of the wound prior to the second step of the surgery.

The second stage of the Masquelet technique was performed 30 days after spacer implantation, following confirmation that the patient’s clinical and biological parameters indicated infection resolution (Figure 7). Blood tests demonstrated the resolution of the inflammatory syndrome, with a white blood cell count (WBC) of 7.65 × 10^3^/µL (normal range: 4.0–10.0 × 10^3^/µL), C-reactive protein (CRP) at 2.5 mg/L (normal range: 0–5.0 mg/L), fibrinogen levels at 5.59 g/L (normal range: 2.0–4.0 g/L), and a platelet count (PLT) of 491 × 10^3^/µL (normal range: 163–337 × 10^3^/µL). On examination, the postoperative incision had healed appropriately, with no clinical signs of infection. Biologically, the inflammatory response had subsided, supporting the decision to proceed with the next surgical stage.

**Figure 8:**
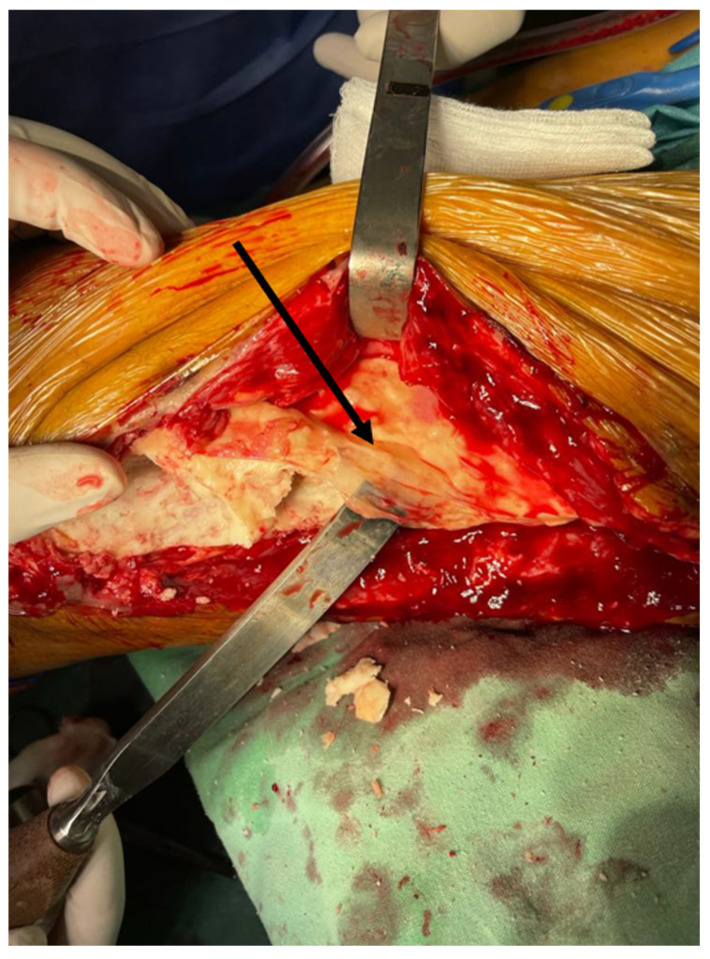
Bioactive membrane presence (arrow).

The second stage involved the removal of the cement spacer and the harvesting of an autologous bone graft to fill the remaining cavity. The process began with a single central longitudinal incision through the self-induced bioactive membrane. Intraoperatively, the membrane appeared white-yellowish, and both the fibrous and well-vascularized structures were visible (Figure 8). Its consistency was firm yet malleable, sufficient to maintain a cavity for the bone autograft. The spacer was removed in smaller fragments using a saw or osteotome.

Damage to the induced bioactive membrane must be avoided in order to maintain an autonomous compartment. The edges of the resected bone should be refreshed using a drill or reamer to remove sclerotic tissue and promote better integration of the graft. Additionally, the medullary canal should be debrided to facilitate communication with the graft. Permanent fixation may be reassessed at this stage if necessary.

**Figure 9:**
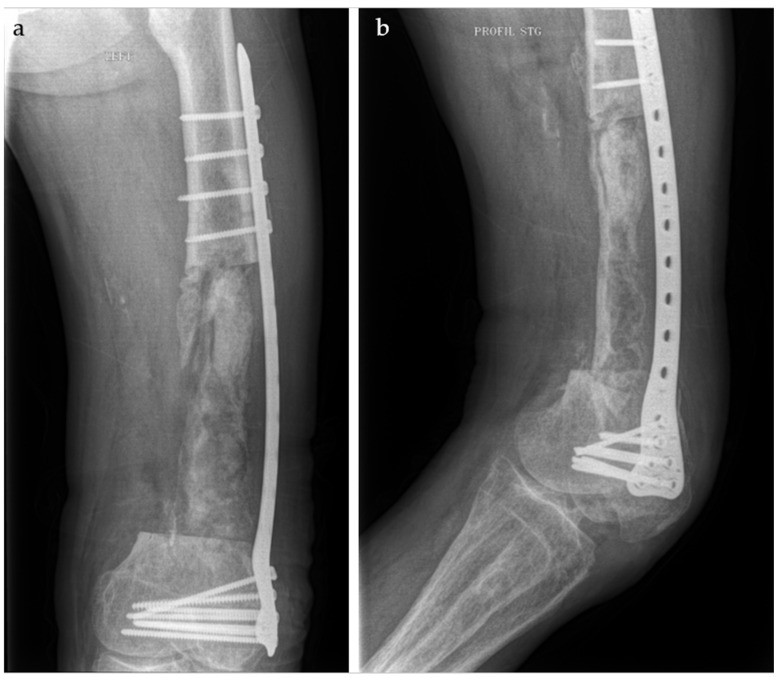
Postoperative radiological aspect. (**a**). antero-posterior view. (**b**). left lateral view.

**Figure 10:**
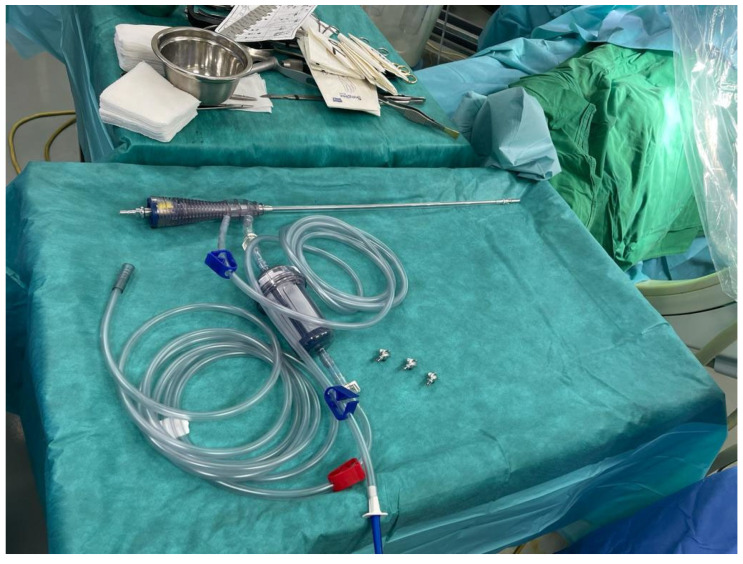
Instrumentation for reaming the medullary canal for bone graft harvesting.

The final step of the intervention involved harvesting the autologous bone graft. Cancellous bone has the ability to form new bone even without mechanical stress; however, it may resorb if the recipient site is poorly vascularized. After harvesting the bone from the iliac crest using specialized instrumentation (Figure 9 and Figure 10), the cavity was filled with the graft, and the wound was then closed in layers. The reamer–irrigator–aspirator (ria) 2 system was utilized to harvest bone graft from the contralateral femur and both tibiae, using progressive reamers ranging from 10 mm to 14 mm. The harvested bone volume was adequate to fill the defect after being combined with NovaBone Putty^®^, a bone substitute manufactured by Novabone, Alachua, FL, USA.

**Figure 11:**
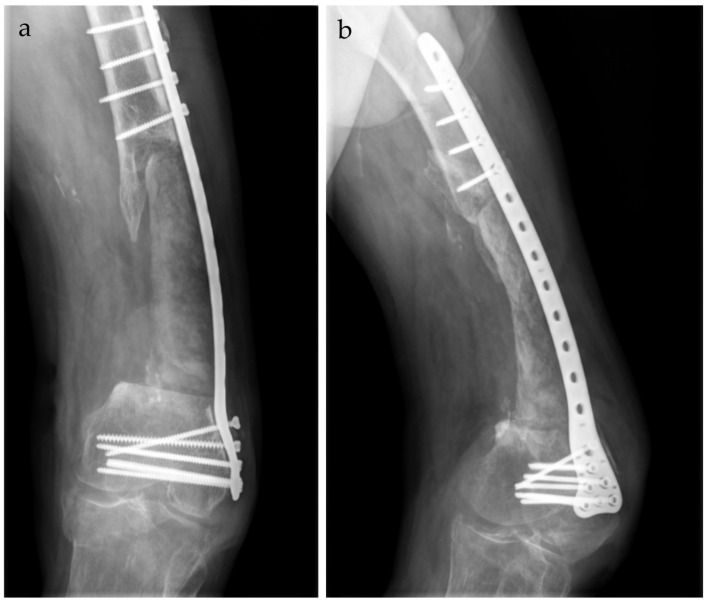
Appearance at six-month postoperative follow-up. (**a**). antero-posterior view. (**b**). left lateral view.

Postoperatively, the patient showed favorable clinical and biological progress, with partial weight-bearing allowed at 3 months after surgery. Figure 11 illustrates the radiological evolution of the patient at 6 months postoperatively.

## 3. Discussion

The induced membrane technique is a relatively new approach for the reconstruction of large bone defects. The formation of the membrane is crucial in this technique, as it possesses several histological and biochemical characteristics that contribute to its success. This case presented significant challenges, but the Masquelet technique demonstrated its advantages.

The Masquelet technique was chosen due to the large bone defect (20 cm), chronic osteomyelitis, and failed non-surgical management, making traditional grafting insufficient. Alternative methods such as vascularized grafts or the Ilizarov technique were considered but presented higher risks and prolonged recovery. This technique was preferred for its ability to induce a bioactive membrane, promote vascularization, and protect the graft from resorption. Success depended on meticulous debridement, infection control, stable fixation, and the patient’s ability to go through staged reconstruction.

The induced membrane is highly vascularized, with the inner layer (facing the cement) resembling the characteristics of a synovial membrane, while the outer layer consists of fibroblasts, myofibroblasts, and collagen. The membrane secretes growth factors, including high concentrations of VEGF (vascular endothelial growth factor) and TGF Beta 1 (Transforming growth factor beta), which have been observed as early as the second week. BMP2 (Bone morphogenetic protein 2) reaches its peak concentration in the fourth week. Studies have shown that extracts from the membrane stimulate bone marrow cell proliferation in vitro. The cancellous bone within the membrane is not subject to resorption. Macroscopic examination of the cross-section of the healed bone graft reveals normal bone anatomy, and the junction between the normal bone and the graft is often difficult to discern during the macroscopic examination of the longitudinal section [22,23,24].

In this case, extensive soft tissue debridement was necessary due to significant necrosis and chronic infection. However, viable tissue was carefully preserved to support the formation of the bioactive membrane. To minimize soft tissue loss, wound coverage strategies were used, ensuring adequate healing before proceeding to the second stage. The successful integration of the bone graft confirms that sufficient soft tissue was preserved to support membrane formation and vascularization, pointing out the importance of balancing infection control with tissue preservation in the Masquelet technique.

The advantages of using the induced membrane technique stem from its function as a biological chamber, which holds the cancellous graft together and prevents its resorption. Additionally, the membrane promotes vascularization and corticalization of the cancellous bone, even in poorly vascularized beds with irradiated tissue or in conditions such as nonunion. The membrane is considered an in situ delivery system for growth factors and osteoinductive factors [25].

An experimental study conducted at the Association of Osteosynthesis Development Institute in Davos demonstrated that the induced membrane technique where the cavity was filled with cancellous bone chips, prevented the resorption of cancellous bone and positively impacted the healing of the autograft [25].

In cases with large bone defects, bone substitutes can be added at a 1:3 ratio without affecting complication rates or healing time compared to reconstruction with cancellous bone alone. The autologous bone graft can also be supplemented with synthetic bone, BMP, bisphosphonates, or hydroxyapatite. However, increased resorption of the autograft has been observed in patients who receive additional local injections of recombinant BMP-7 [26,27].

The original fixation method for stabilizing bone after excision was external fixation, although internal fixation can also be utilized [26]. Depending on the fixation method, variations in technique may have different effects on membrane formation. In an experimental study by Kaneko et al., several fixation methods were tested on mice. This study successfully introduced a mouse plate fixation model for Masquelet’s induced membrane technique (MIMT), offering a valuable tool for evaluating induced membrane formation. Specifically, the plate-fixed Masquelet (P-M) and intramedullary-fixed Masquelet (IM-M) groups exhibited distinct membrane structures and macrophage responses. The IM-M group demonstrated more vigorous bone growth and thicker membranes compared to the P-M group. These results suggest that the fixation method significantly influences the biological environment within the induced membrane, providing important insights that could aid in optimizing MIMT for clinical applications [28].

The time required to achieve full weight-bearing is influenced by both the fixation method and the extent of the bone defect. A study by Frese et al. found a significant correlation between defect length and the time to full weight-bearing (*p* = 0.0134) [29].

The risk of fracture in regenerated bone following the Masquelet technique is an important consideration, particularly in large defects where structural integrity is gradually restored [30,31]. Although the induced membrane promotes bone healing and vascularization, the new bone remains susceptible to mechanical stress, especially in the early phases of consolidation. To reduce this risk, stable fixation methods, gradual weight-bearing progression, and regular radiographic monitoring are essential [32,33].

With advancements such as hydroxyapatite-coated external fixation pins, locked intramedullary nails, and locking plate systems, immediate weight-bearing is now possible. However, careful consideration must be given to defect size and other factors that may impact healing and increase fracture risk [34,35,36,37]. While early mobilization promotes faster recovery and plays a vital role in mitigating the physical and psychological challenges faced by patients with prolonged disabilities, the risk of fracture in regenerated bone remains higher. As a result, a gradual or even delayed weight-bearing approach is often recommended [38,39].

The Masquelet technique is a valuable approach for segmental bone loss, promoting bone regeneration through an induced membrane that improves vascularization, contains growth factors, and prevents fibrous tissue invasion [40]. However, its effectiveness depends on adequate vascularization, as growth factors function only in well-vascularized tissues. Poor vascularization may compromise healing, and factors such as fixation methods, timing of the second stage, and bone substitute integration can influence the outcomes [31]. The two-stage process requires prolonged treatment, causing challenges for some patients. A personalized, closely monitored approach is essential for optimizing healing and clinical success [41,42].

Future advancements in the Masquelet technique should focus on optimizing spacer materials, optimizing bone graft integration, and exploring single-stage procedures to improve efficiency. Research into alternative biomaterials, such as titanium, silicone, and calcium sulfate, has shown promise in generating osteogenic membranes with improved properties [43]. Additionally, the incorporation of synthetic scaffolds and bioactive additives may further support bone regeneration [44,45,46]. These innovations have the potential to make treatment more effective, less invasive, and more accessible, particularly in settings with limited resources.

## 4. Conclusions

This bone grafting technique, which utilizes a biologically induced membrane created by placing an antibiotic-impregnated cement spacer, provides a logical and effective alternative for reconstructing complex distal femur fractures with significant bone defects. The technique has shown reproducible results with a high success rate.

## Data Availability

The data presented in this study are available upon reasonable request from the corresponding author.

## Abbreviations

BMP	Bone morphogenetic protein
CRP	C-reactive protein
CT	Computer tomography
ESR	Erythrocyte sedimentation rate
Hb	Hemoglobin
MCHC	Mean corpuscular hemoglobin concentration
MIMT	Masquelet’s induced membrane technique
MSSA	Meticilin-sensitive *Staphylococcus aureus*
PLT	Platelets
PMMA	Polymethylmethacrylate
TGF Beta 1	Transforming growth factor beta
VEGF	Vascular endothelial growth factor
WBC	White blood cell
